# Relapsed/Refractory Chronic Lymphocytic Leukemia Patients Treated with Fixed Duration Venetoclax-Rituximab: Assessment of Response with Ultrasound, and Relationship with Minimal Residual Disease

**DOI:** 10.3390/jcm12051772

**Published:** 2023-02-23

**Authors:** Edoardo Benedetti, Claudia Baratè, Fabrizio Mavilia, Emilia Bramanti, Riccardo Morganti, Valentina Guerri, Giulia Cervetti, Enrico Capochiani, Ilaria Bertaggia, Salvatore Massimo Stella, Ginevra Traverso, Benedetto Bruno, Sara Galimberti

**Affiliations:** 1 Azienda Ospedaliero Universitaria Pisana, Hematology Unit, Department of Clinical and Experimental Medicine, University of Pisa, 56126 Pisa, Italy; 2 Italian School of Basic and Emergency Ultrasound (SIUMB), 56100 Pisa, Italy; 3 Institute of Chemistry of Organometallic Compounds (ICCOM), Italian National Research Council (CNR), Via G Moruzzi 1, 56124 Pisa, Italy; 4 Azienda Ospedaliero Universitaria Pisana, Section of Statistics, 56126 Pisa, Italy; 5 Hematology Unit, Azienda USL Toscana Nord Ovest, 57124 Livorno, Italy; 6 Department of Molecular Biotechnology and Health Sciences, University of Turin, 10126 Torino, Italy

**Keywords:** chronic lymphocytic leukemia, ultrasound sonography, venetoclax-rituximab

## Abstract

A fixed duration of venetoclax-rituximab (VenR) resulted in a significant benefit of both PFS and in the attainment of an undetectable minimal residual disease (uMRD) compared with bendamustine-rituximab in relapsed/refractory (R/R) chronic lymphocytic leukemia (CLL) patients. The 2018 International Workshop on CLL guidelines, outside the context of clinical trials, suggested ultrasonography (US) as a possible imaging technique to evaluate visceral involvement, and palpation to evaluate superficial lymph nodes (SupLNs). In this real-life study we prospectively enrolled N = 22 patients. Patients were assessed by US, to determine nodal and splenic response in R/R CLL patients treated with a fixed duration VenR. We found an overall response rate, complete remission, partial remission, and stable disease, of 95.4%, 68%, 27.3%, and 4.5%, respectively. Responses were also correlated with risk categories. The time to response, and the time to clearance of the disease in the spleen, in abdominal LN (AbdLNs), and in SupLNs were discussed. Responses were independent from LN size. The correlation between response rate with MRD were also investigated. US allowed to detect a substantial CR rate correlated with uMRD.

## 1. Introduction

Chronic lymphocytic leukemia (CLL) is the most common adult leukemia in Western countries and remains largely incurable despite recent treatment options [1,2,3]. Allogenic stem cell transplantation is unsuitable for most patients [4]. Historically the standard of treatment has been chemoimmunotherapy (CIT) that achieves disease control and prolongs survival [5]. Recently novel targeted therapies in first line or in relapsed/refractory (R/R) CLL patients have improved outcome over CIT [5]. Targeted therapies comprise Bruton tyrosine kinase [BTK] inhibitors, ibrutinib and acalabrutinib, and the phosphatidylinositol 3-kinase [PI3K] inhibitors, idelalisib and duvelisib [5]. The anti-apoptotic protein BCL2 is overexpressed in CLL cells [6,7], and it represents a therapeutic target. Venetoclax is a highly selective potent BCL2 inhibitor that acts regardless of the presence of *TP53* mutation. The combination of venetoclax with rituximab (anti-CD20 antibody) has shown to overcome microenvironment-induced resistance to venetoclax [8]. Rituximab can obtain clearance of minimal residual disease (MRD). In patients treated with CIT, undetectable minimal residual disease (uMRD, <1 CLL cell per 10^4^ leucocytes) is linked to more favorable overall survival (OS) and progression free survival (PFS) [9].

In the MURANO study a fixed duration venetoclax plus rituximab (VenR) has resulted in improved PFS and OS compared to bendamustine plus rituximab (BR) treatment in patients with R/R CLL [3]. Investigators have found 93.3% overall response rate (ORR) with 26.8% complete remissions (CR) or CR with incomplete hematological recovery (CRi). After 9 months of treatment the clearance of MRD in peripheral blood (PB) was achieved in 62.4% of patients [6].

The 2018 International Workshop on Chronic Lymphocytic Leukemia (IWCLL) guidelines [9] suggested ultrasonography (US) as a possible imaging technique to evaluate visceral involvement, and palpation to evaluate superficial lymph nodes (SupLNs) in general practice [9]. Ultimate ultrasonography machines are equipped with high resolution linear probes, and US features of normal and diseased SupLNs have been published [10,11,12,13]. Recently, US features of nodal and splenic involvement by CLL has been described [14], and US has been used to assess nodal and splenic response to venetoclax therapy [15].

This study is aimed to assess with US, nodal and splenic response in R/R CLL patients treated with a fixed duration VenR, and to compare the US results with MRD response.

The endpoints of this real-life study are the evaluation of (i) the response rates ORR, CR, partial remission (PR), stable disease (SD), and progressive disease (PD); (ii) the time to response; (iii) the response rate of the LNs with the longest diameter < 5 cm vs. ≥5 cm [16,17]; (iv) the correlation of nodal and splenic response with PB-MRD.

## 2. Materials and Methods

This study was conducted in the Hematology Unit of the University of Pisa, Italy, and all patients signed a written consent form (Ethical Committee approval Nr. ID22968, 22 September 2022). We prospectively enrolled N = 22 R/R CLL patients.

The diagnosis of CLL and the definition of R/R patients was made according to the 2018 International Workshop on Chronic Lymphocytic Leukemia guidelines [9]. After 5 weeks ramp-up, all patients received a fixed duration VenR treatment in accordance with what previously described [8]. In detail, venetoclax was administered with a 5-week schedule of gradual increase in the dose (ramp-up) from 20 mg per day to 400 mg per day. After completion of the ramp-up period for venetoclax, administration of rituximab was initiated at 375 mg/m^2^ for the first dose (day1 of cycle 1), and 500 mg/m^2^ thereafter (day 1 of cycle 2 through cycle 6) in 28-day treatment cycles, while administration of venetoclax was continued. Patients received a total of six 28-day cycles of VenR, followed by single-agent venetoclax (400 mg) once daily for a total of 2 years. The 2-year venetoclax treatment period was calculated from day 1 of cycle 1 after venetoclax dose ramp-up [6].

The US assessment was performed for each patient using a General Electric (GE) Logiq e10s ultrasound sonographer. A 2–5 MHz bandwidth GE convex probe was used for abdominal scanning, and a linear matrix array GE probe bandwidth 4–16 MHz was used for SupLN assessment. The physician who performed the US examinations, was a hematologist member and teacher at the Italian School for basic and emergency ultrasound (SIUMB) of the University of Pisa with more than 20 years’ experience in lymphoproliferative disorders and US [14,15].

The anatomical superficial regions assessed with US were following: cervical, axillary, inguinal, supraclavicular, and subclavicular, bilaterally. For each superficial anatomical region, the parameters assessed with US were the number of pathological LNs, their dimensions (longitudinal diameter in mm), the echostructure and vascular pattern [12,14,15,18,19,20,21]. We also assessed the echostructure of each LN and in particular: (i) shape, (ii) the presence of sharp borders [21,22], (iii) the long axis/short axis ratio (L/S) [19,21,22], (iv) the presence or absence of vascular hilum and its shape displaced, or truncated, or compressed narrowed hilum [21,22], (v) cortical morphology, asymmetrically thickened cortex, eccentrical cortical hypertrophy [21,22], cortical reticulum [19,21], (vi) vascular pattern such as hilar, mixed subcapsular and hilar, and chaotic [12,19].

Using US, SupLNs were defined as either pathological (CLL involvement) or non-pathological either reactive or liposclerotic as previously described [12,14,15,20]. Echostructural features of SupLNs involved by CLL have been also previously described [14]. The spleen was assessed for each patient describing both splenic longitudinal diameter, and the cross-sectional area as previously reported [13,14,15,20,23]. It was recently published that CLL may have five US patterns of splenic involvement: (i) homogeneous splenomegaly, (ii) finely inhomogeneous diffuse infiltration, (iii) micronodular diffuse infiltration, (iv) micronodular disperse hypoechoic lesions, (v) macronodular involvement [14].

In this study the spleen has been defined in complete response only when it fulfilled both the dimensional criteria (longitudinal diameter and cross-sectional area) [14,15,20,23], and the attainment of a normal splenic echostructure as previously described [14]. In AbdLNs the echostructure cannot be defined as for SupLNs [12], thus the definition of the response is based only on their dimensions (LNs < 1.5 cm) [9]. When patients were enrolled in the study a total of N = 561 SupLNs and N = 86 AbdLN were analyzed with US before treatment was initiated (T0, baseline). For each patient a median of N = 28 (range 12–32) SupLNs have been scanned with US. Both the number and the echostructure of each LN were recorded and were checked at every following US assessment. If a further US assessment was necessary (for example if treatment had to be temporarily discontinued for any causes) all the SupLNs and AbdLNs found were analyzed before the treatment was resumed. US assessment of each patient was performed at the time points described in Table 1.

At each US assessment time point, patients were also assessed for PB-MRD status with flow cytometry (Table 1) [2,9]. Undetectable MRD was defined as below the threshold of one tumor cell per 10^4^ white cells [6]. We analyzed PB-MRD because it was shown to be a surrogate of bone marrow MRD as previously described [6]. If a patient discontinued or temporarily discontinued the treatment, the interval of discontinuation was recorded, and US was repeated the day the treatment was re-initiated, and US and MRD follow up assessment continued at each of the time points established at baseline, without any change in the schedule of follow up.

### Statistical Analysis

Raw data of the population characteristics are shown in Table 2 and the categorical ones were described by absolute and relative (%) frequency. To analyze categorical data, a chi square test and z-test for two proportions were performed. Significance was fixed at 0.05 and all analyses were carried out with the SPSS v.28 technology.

## 3. Results

### 3.1. Patients Characteristics

From December 2021 to July 2022 N = 22 R/R CLL patients were prospectively enrolled in this study. Patients’ characteristics, and risk categories are listed in Table 2. Number 17/22 (77.3%), N = 1/22 (4.5%), and N = 4/22 (18.2%) patients previously received N = 1, N = 2, and N = 3 lines of therapy, respectively.

### 3.2. Response

**Figure 1:**
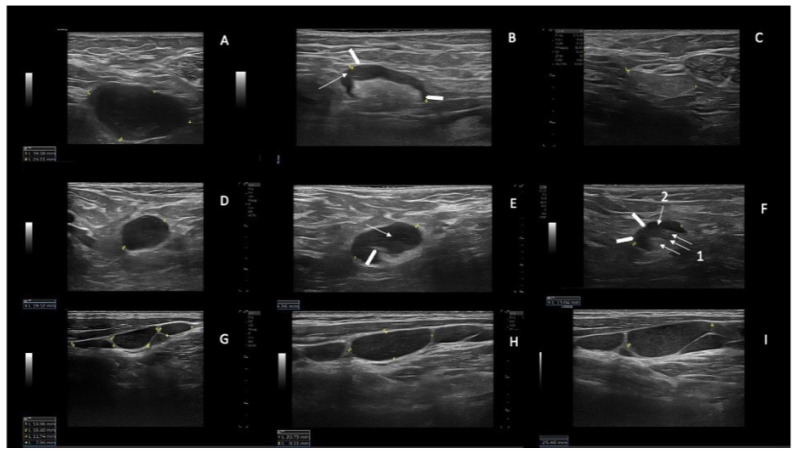
Examples of SupLNs achieving CR, SupLNs in persistent PR, and SupLN in SD. (**A**) Right axillary CLL-LN (39 mm × 24 mm) at baseline (T0). The LN appears hypoechoic, with sharp and regular borders, without a visible hilum, L/S ratio < 2, and with a thickened and reticulated cortex. (**B**) the same LN at T3 (27.3 mm) showing a partial involvement by CLL. The cortex appears inhomogeneous (the anterior part more thickened than the posterior part-white arrowheads, with reticulated cortex-white arrow). The hyperechoic hilum is visible, although irregular. (**C**) the same LN measuring 19.3 mm, has become liposclerotic (nodal CR) at T6. (**D**) Right axillary CLL-LN (19 mm) at T0, with round shape (L/S < 2) showing the same US features as for the LN shown in panel A. (**E**) the same LN at T6 showing a partial involvement by CLL (19.2 mm): US show a visible hilum which is displaced and truncated (white arrowhead 1), the cortex is thickened inhomogeneous, with reticulation (white arrow). (**F**) the same LN at T15 (13 mm) showing a persistent partial CLL involvement of the LN: the cortex is still not homogeneously thickened, which determines irregular borders of the LN (white arrowheads) and irregular shape of the hilum (white arrow 1) which appears dislocated in the posterior part of the LN. The cortex shows reticulation (white arrow 2). (**G**) left laterocervical CLL-LN in SD at T0. (**H**) left laterocervical CLL-LN in SD at T12. (**I**) Left laterocervical CLL-LN in SD at T24. The SupLNs present “chain”-shaped, contiguous, sharp borders without US visible hilum. The cortex is inhomogeneous and thickened, with reticulation.

With a median follow up of 12 months (range 6–24 months), we found a nodal (SupLNs and AbdLNS) and splenic response in N = 21/22 patients with 95.4% ORR. We found N = 15/22 (68%) CR, N = 6/22 PR (27.2%), and SD N = 1/22 (4.5%) (Figure 1). We did not detect PD during treatment.

**Figure 2:**
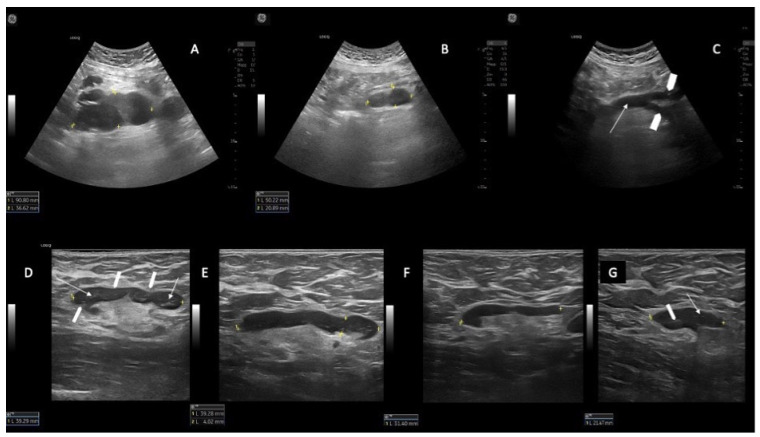
Example of AbdLN in CR but with persistence of CLL diseased SupLN (PR) in the same patient. (**A**) AbdLNs (longitudinal US scan with a convex probe) at baseline (T0) (90.1 mm). (**B**) the same AbdLN at T3 (50.2 mm). (**C**) disappearance of AbdLN (CR) at T6. The abdominal aorta is shown in a longitudinal scan (white arrow) and the right and left iliac arteries are visible (white arrowheads). (**D**) example of a right axillary CLL-SupLN at T0 (39.2 mm); the LN has an oval shape (long axis/short axis (L/S) > 2), the cortex is thickened, inhomogeneous, and reticulated (white arrows), which determines the lobular profile of the LN (white arrowheads). (**E**–**G**) the same right axillary CLL-SupLN at T6 (39.2 mm) (**E**), T12 (31.4 mm) (**F**) and T18 (21.5 mm) (**G**). In panel G there is still a partial involvement by CLL: the LN has oval shape (L/S > 2), the cortex is thickened and inhomogeneous and reticulated (white arrow), and the posterior part more thickened than the anterior part). The hilum is visible but is compressed and dislocated by the thickened cortex.

In patients whose response at time of the US assessment was PR (N = 6/22), we found that if the spleen and AbdLNs were pre-treatment involved, they obtained CR. Nevertheless, patients were considered in PR because of the persistence of superficial CLL diseased LNs (Figure 2).

**Figure 3:**
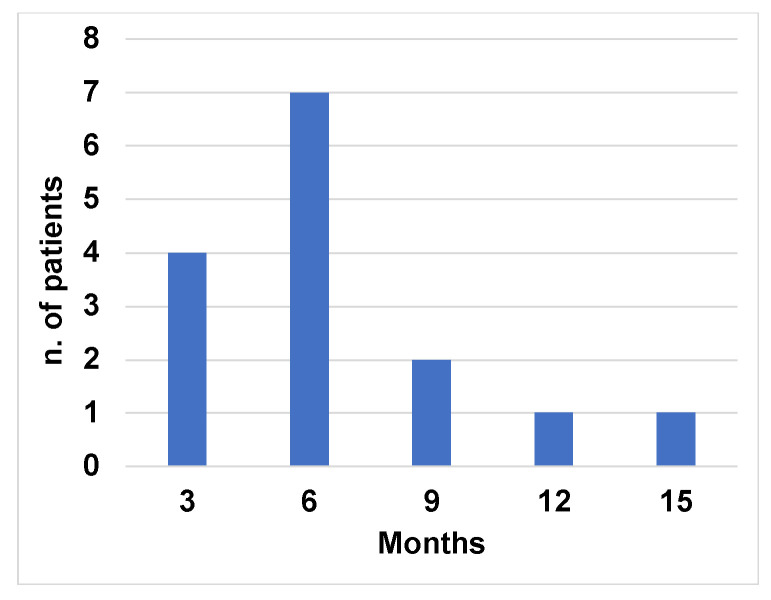
Frequency distribution of patients achieving CR at different time point of US follow up.

Figure 3 shows the time to CR. We found that in patients who obtained nodal and splenic CR (N = 15), N = 13/15 (86.7%) patients reached a CR by 9 months from the beginning of the treatment.

We analyzed if the risk categories of our study population were associated with the quality of nodal and splenic response. We found that IGHV mutational status had no impact on the attainment of nodal and splenic CR (*p* = 0.717). Nevertheless, we found that patients without Del(17) or *TP53* mutated had a higher percentage of CR, with a *p*-value showing a trend toward a statically significant difference (*p* = 0.147, and *p* = 0.101, respectively).

### 3.3. Correlation of Nodal and Splenic Response with PB-MRD Response

Fifteen out of 15 patients who attained nodal and splenic CR and two patients in PR, reached uMRD at a median time of 3 months (range 3–15 months). Overall N = 17/21 (80%) patients reached uMRD.

### 3.4. Time to Response

**Figure 4:**
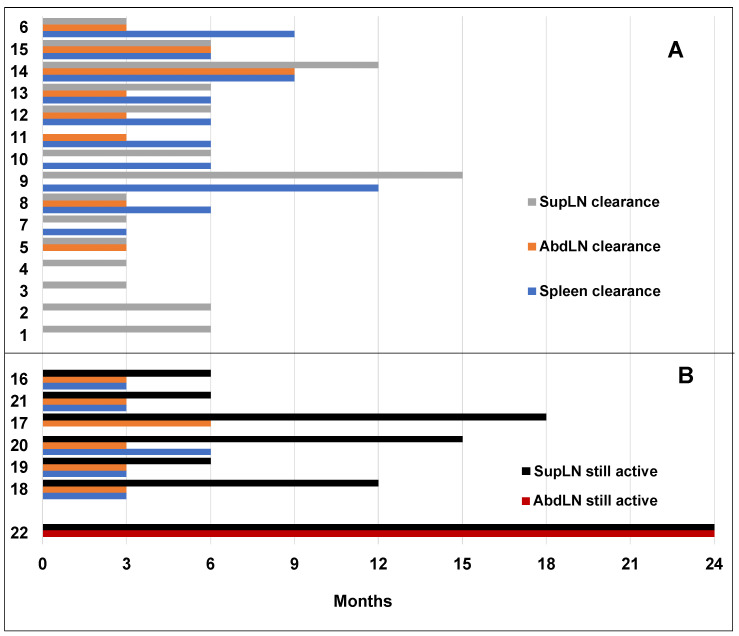
Plot of AbdLNs and SupLNs achieving CR or still diseased, and splenic response in 22 patients examined. (**A**) Patients who achieve CR (AbdLNs, SupLNs and splenic CR). (**B**) Lymphnodal and splenic US response in patients in PR and in the patient in SD. In patients in PR, although AbdLNs and splenic response achieved a CR, SupLNs were still diseased by CLL. Patient 22 was in SD at T24 and both AbdLNs and SupLNs were still involved by CLL.

We evaluated the median time to CR of the spleen, of AbdLNs, and SupLNs. We found that it was 6 months (range 3–12 months), 3 months (range 3–9 months), and 6 months (range 3–15 months), respectively (Figure 4).

### 3.5. Lymphnodes Dimensions and Response

**Figure 5:**
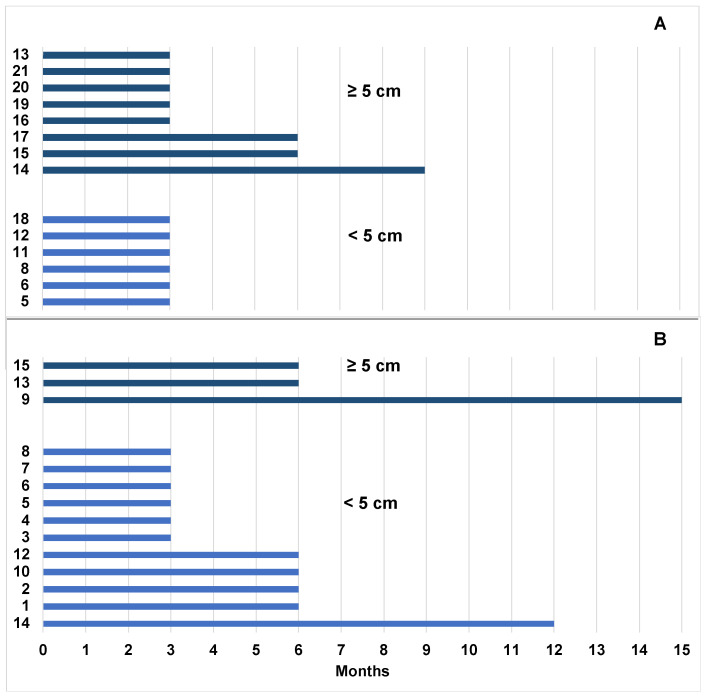
Time to response of LN< 5 cm vs. ≥ 5 cm. (**A**) Patients with AbdLN involvement before the initiation of treatment having LN < 5 cm reached CR at T3; patients having LN ≥ 5 cm, reached CR at T3 (N = 5 pts), at T6 (N = 2 pts), and T9 (N = 1 pts). (**B**) Time to CR of SupLNs < 5 cm (N = 14/15 patients) was T3 (N = 6 pts), T6 (N = 4 pts) and T12 (N = 1 pts). If SupLNs were ≥ 5 cm time to CR was T6 (N = 2 pts), and T15 (N = 1 pts).

We found that dimensions of LNs < 5 cm or ≥ 5 cm did not impact on the probability of achieving CR (Figure 2A–C). We found that 94.1% of patients with LNs < 5 cm and 81.8% with LNs ≥ 5 cm reached CR at T ≤ 6 months, respectively (*p*-value = 0.688). For T > 6 months, 5.9 % of patients with LNs < 5 cm and 18.2 % with LNs ≥ 5 cm reached CR, respectively (*p*-value = 0.688) (Figure 5A,B).

### 3.6. Treatment Discontinuation

Four patients had a temporary discontinuation of VenR during the combination treatment phase, for 3.5, 1.5 and 3 months because of SARS-CoV-2 infection and for 16 days because of febrile neutropenia. An additional US assessment of these patients was performed right before the treatment was reintroduced, and it was found that that the quality of response to treatment (N = 3 CR and N = 1 PR) was unchanged despite discontinuation, without any US detectable PD.

### 3.7. Additional Observations

Using a high resolution US linear probe, we found that in several SupLNs the echostructure showed features of involvement by CLL present only in a small portion of the LN, which was never described before. This echostructure feature was detected in N = 8 patients (36.3% of our casuistic), and in 4/8 it was a transient US status, because eventually achieved a status of US nodal CR (Figure 1A–C), and accordingly reached a PB-uMRD. Two patients out of 4, with a partial US residual involvement by CLL (Figure 1D–F and Figure 2D–G) attained uMRD.

## 4. Discussion

CLL remains a malignancy largely incurable despite advances in new treatment options [5]. In R/R patients, BTKi ibrutinib is an effective therapeutic option [24], but it requires extended administration until unacceptable toxicity or disease progression. Fixed duration therapies may achieve deep and durable clinical responses and may reduce the development of resistant sub clones [25].

Following the 2018 International Workshop on Chronic Lymphocytic Leukemia guidelines [9], CT scan is not recommended outside the context of clinical trials because potentially harmful [9]. US features of normal and diseased SupLNs (metastatic, lymphoproliopherative disorders and CLL) have been published [12,13,14,15,26].

The achievement of CR with respect to PR has been shown to correlate with the duration of response and to lead to a longer OS [27]. Seymour et al. reported ORR and SD in 93.3% and 2.1% of patients, respectively [6]. In this study we found ORR in 95.4% and SD in 4.5% of patients without statistically significant difference between the two studies (*p*= 0.938 for ORR and *p* = 0.969 for SD). In our real-life study we found that 68% of patients reached nodal and splenic CR.

In IWCLL 2018 guidelines nodal CR is defined in clinical practice as the absence of significant lymphoadenopathy by physical examination [9]. The definition of nodal CR with US of SupLNs takes into account multiple echostructure parameters [10,11,12,13,14,21,24]. Recently, in a prospective study it was shown that US could detect a number of CLL diseased SupLNs higher than palpation, and US could size SupLNs more accurately than palpation [14]. Moreover, it was shown that CLL-SupLNs could become lipoplastic LNs reaching CR during venetoclax therapy, although their dimensions still excided 15 mm [15].

The levels of MRD were shown to be highly predictive of duration of remission after veneteoclax cessation [28]. Seymour et al. in their study reported 62.4% uMRD, and, accordingly, we found uMRD in 80% of patients examined without a statistically significant differences (*p* = 0.162) [6]. Kater et al. showed that MRD had no effect on PFS in patients in CR/CRi, all having either uMRD or low MRD positivity [28]. Thus, the attainment of CR is of valuable importance.

In our study, we found a high correlation between nodal and splenic CR and uMRD. All patients who achieved nodal and splenic CR also achieved uMRD at median time of 9 months (Figure 3 and Figure 4). We found a correlation between the depth of response with Del(17) and *TP53* mutation, but not with IGHV mutational status, according to what previously described [6,28]. However, in patients with Del(17) and *TP53* mutation we found only a trend toward a statistical significance probably due to the sample size [6,28].

Wierda et al. [29] and O’Brien et al. [17] showed that patients with LN dimension ≥5 cm have a lower PFS and reached less CR than patients with LN dimension <5 cm. In our study we did not find a correlation between LNs size (LNs < or ≥ 5 cm) and the probability of patients either to reach nodal CR nor a correlation with the time to CR (Figure 5). An extended follow up of our study population will show if patients with LNs ≥ 5 cm will experience a higher relapse risk.

Moreover, we report on SupLNs with echostructure consistent with a partial involvement by CLL detected with high resolution linear probe. This partial involvement of SupLNs has never been described before in patients affected by CLL. These echostructure features appear to either stabilize into a persistent PR (Figure 1D–F and Figure 2D–G) or appear a transient status turning into a complete nodal CR (Figure 1A–C). This transient change in the echostructure of SupLNs might be explained by a total of ten US examinations (1 at baseline T0, three during combination treatment and six from EoCT until the end of second year).

Mato et al. [30] showed that treatment interruption, which mostly occurred during the combination treatment phase, had not statistically significant impact on clinical outcome. In our study, according to Mato et al., four patients temporarily discontinued the treatment during the combination phase without changes of the quality of response achieved.

In conclusion, to the best of our knowledge, in this real-life study we have assessed for the first time in general practice the responses to a fixed dose VenR in R/R CLL patients by US. Ultrasound detected echostructural changes consistent with nodal and splenic CR in a substantial proportion of patients, along with the achievement of uMRD. US has allowed us to follow up patients with frequent time points during the 2-year study period due to the absence of radiation hazard. In a real-life scenario of response assessment of CLL patients, US could be a useful tool and an added value to support palpation.

## Figures and Tables

**Table 1:** Definition of study timepoints and timing of analysis methods.

Time Points	Definition and Analysis Methods		
Baseline	US assessment (abdomen+ superficial LNs)	PB-MRD	PE, CBC, and CLEx
Day 1 cycle1	US assessment (abdomen+ superficial LNs)		PE+ CBC
Day 1 cycle 2			PE+ CBC
Day 1 cycle 3			PE+ CBC
Day 1 cycle 4	US assessment (abdomen+ superficial LNs)	PB-MRD	PE+ CBC + CLEx
Day 1 cycle 5			PE+ CBC
Day 1 cycle 6 (EoCT)	US assessment (abdomen+ superficial LNs)	PB-MRD	PE+ CBC + CLEx
Every three months after EoCT until EoT Months 9, 12, 15, 18, 21, and 24	US assessment (abdomen+ superficial LNs) (Months 9, 12, 15, 18, 21 and 24)	PB-MRD (Months 9, 12, 15, 18, 21, and 24)	PE + CBC+ CLEx (Months 9, 12, 15, 18, 21 and 24)

PE = Physical examination, PB-MRD = peripheral blood MRD; CLEx = complete laboratory examination; CBC = complete blood count; EoCT = end of combination treatment.

**Table 2:** Patients’ characteristics.

All Patients	Total No.	Time from Last Treatment to Venetoclax-Rituximab
Age		
<65	9	
≥65	13	
No. Previous therapies		
1	17	-
2	1	-
≥3	4	-
Type of prior therapies		
BR	11	Median 18.6 months (range 3–84 months)
R-CHL	2	3 months
FCR (1st line), R-CHL (2nd line), IBRU (3rd line)	4	Median 12 months (range 6–15 months)
IBRU	2	12 months and 15 months
FCR	2	117 months and 146 months
BR (1st line), R-CHL (2nd line)	1	8 months
Chromosome 17p deletion status		
Absent	16	
Present	5	
NA	1	
TP53 mutation status		
Unmutated	14	
Mutated	7	
NA	1	
Baseline IGHV mutation status		
Unmutated	16	
Mutated	5	
NA	1	

BR = bendamustine plus rituximab; R-CHL = rituximab plus Chlorambucil; FCR = fludarabine, cyclophosphamide and rituximab; IBRU = ibrutinib. Del (17p) = 17p deletion identified by fluorescence in situ hybridization in PB [6]; IGHV = immunoglobulin heavy-chain variable gene mutation status at the beginning of the treatment.

## Data Availability

The datasets generated and analyzed during the current study are available from the corresponding author on reasonable request.
